# Correction

**DOI:** 10.5195/jmla.2017.128

**Published:** 2017-01

**VOLUME 104**

**104(3) July, page 242**

Bramer WM, Giustini D, de Jonge GB, Holland L, Bekhuis T. De-duplication of database search results for systematic reviews in EndNote. J Med Libr Assoc. 2016 Jul;104(3):240–3. DOI: http://dx.doi.org/10.3163/1536-5050.104.3.014.

Page 242: Table 1, row C3, should state:

Review the top references without page numbers and those with page numbers starting with the number 1 for equivalent author names.

Only articles without page numbers or page numbers starting with 1 should be reviewed.

